# Peptide Functionalized Gold Nanorods for the Sensitive Detection of a Cardiac Biomarker Using Plasmonic Paper Devices

**DOI:** 10.1038/srep16206

**Published:** 2015-11-10

**Authors:** Sirimuvva Tadepalli, Zhifeng Kuang, Qisheng Jiang, Keng-Ku Liu, Marilee A. Fisher, Jeremiah J. Morrissey, Evan D. Kharasch, Joseph M. Slocik, Rajesh R. Naik, Srikanth Singamaneni

**Affiliations:** 1 Institute of Material Science and Engineering and Department of Mechanical Engineering and Material Science, Washington University in St. Louis, St. Louis, MO, 63130, USA; 2 Soft Matter Materials Branch, Materials and Manufacturing Directorate, Wright Patterson Air Force Base, Dayton, OH 45433, USA; 3 Department of Anesthesiology, Division of Clinical and Translational Research, Washington University in St. Louis, St. Louis, MO 63110, USA; 4 Siteman Cancer Center, Washington University in St. Louis, St. Louis, MO 63110, USA; 5 Department of Biochemistry and Molecular Biophysics, Washington University in St. Louis, St. Louis, MO 63110, USA

## Abstract

The sensitivity of localized surface plasmon resonance (LSPR) of metal nanostructures to adsorbates lends itself to a powerful class of label-free biosensors. Optical properties of plasmonic nanostructures are dependent on the geometrical features and the local dielectric environment. The exponential decay of the sensitivity from the surface of the plasmonic nanotransducer calls for the careful consideration in its design with particular attention to the size of the recognition and analyte layers. In this study, we demonstrate that short peptides as biorecognition elements (BRE) compared to larger antibodies as target capture agents offer several advantages. Using a bioplasmonic paper device (BPD), we demonstrate the selective and sensitive detection of the cardiac biomarker troponin I (cTnI). The smaller sized peptide provides higher sensitivity and a lower detection limit using a BPD. Furthermore, the excellent shelf-life and thermal stability of peptide-based LSPR sensors, which precludes the need for special storage conditions, makes it ideal for use in resource-limited settings.

Plasmonic biosensors based on localized surface plasmon resonance (LSPR) are highly attractive for lab-on-chip devices that are cost-effective and used in point-of-care biodiagnostics^1^. LSPR of metal nanostructures is shown to be sensitive enough to differentiate various inert gases (refractive index difference on the order of 3 × 10^−4^ refractive index units (RIU)), probe the conformational changes of individual biomacromolecules, detect single biomolecule binding events, monitor the kinetics of catalytic activity of single nanoparticles and even optically detect a single electron^2^,^3^,^4^,^5^,^6^. In the design of LSPR-based biosensors, two factors are of prime importance: (i) the bulk refractive index sensitivity and the electromagnetic decay length of the nanostructures employed as optical transducers. There have been numerous studies that focus on the design, synthesis and the validation of novel plasmonic nanostructures with high bulk refractive index sensitivity^7^,^8^,^9^. However, studies focusing on understanding the effect of the electromagnetic (EM) decay length on the ultimate performance of the LSPR-based biosensor are limited^10^,^11^. Although the decay in the sensitivity of plasmonic nanostructures with distance has been widely investigated^12^,^13^, to the best of our knowledge, a direct comparison of the sensitivity of LSPR biosensors based on recognition layers of different sizes (i.e., thickness of the recognition layer) is largely unexplored.

Specific biomolecular interactions such as antibody-antigen interactions form the basis for numerous bioassays including enzyme-linked immunosorbent assay (ELISA), immunoblotting, and immunoprecipitation assays^14^,^15^,^16^. So far, most of the plasmonic biosensors have relied on antibodies as target recognition elements due to the selectivity and sensitivity of antibodies^17^. Although antibodies offer excellent molecular recognition capabilities, they do suffer from: (i) limited pH and temperature stability; loss of conformation and recognition functionality in non-aqueous media; (iii) high cost associated with generating antibodies; and (iv) poor compatibility with micro and nanofabrication processes for efficient integration with various transduction platforms.

Owing to the evanescent nature of the EM field at the surface of the plasmonic nanostructures, the LSPR wavelength shift exhibits a characteristic decay with the increasing distance from the surface of the nanotransducer, given by,


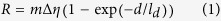


where *R* is the LSPR shift, *m* is the bulk refractive index sensitivity (RIS), Δ*η* is the difference in the refractive index between the adsorbed layer and the surrounding medium, *d* is the layer thickness and *l*_*d*_ is the EM decay length^12^. Thus the LSPR shift measured upon a binding event depends on the RIS and the decay length, which are characteristic to a given nanotransducer. The large size of natural antibodies (~150 KDa) can significantly lower the sensitivity of LSPR-based biosensors in which the sensing volume (typically characterized by the EM decay length from the surface of the transducer) is relatively small compared to SPR-based sensors^18^. Clearly, these considerations highlight the need for alternate recognition elements that exhibit high specificity and stability to translate LSPR-based biosensors to point-of-care diagnostics in resource-limited settings. In this paper, we describe a bioplasmonic paper device (BPD) for LSPR-based bioassay wherein the plasmonic nanostructures are functionalized with a peptide recognition elements with high affinity for the cardiac biomarker troponin I (Fig. 1).

One out of every four deaths in the United States is related to heart disease, which is the leading cause of death in both men and women. Coronary heart disease, which leads to myocardial infarction, is the most common type of heart disease killing 380,000 people annually^19^. It is widely accepted that the concentration of troponin (cTnI) in blood serum serves as a highly sensitive and specific biomarker for the detection of myocardial damage and for risk stratification in such patients (clinical range of 0.1 to 10 ng/ml in blood)^20^. Troponin is also considered to be an important biomarker for muscular fatigue and hypoxia^21^. Unfortunately, existing immunoassays require clinical lab conditions for the quantitative detection of troponin in physiological fluids. Apart from the conventional immunoassays such as ELISA, there have been recent reports that demonstrate plasmonic biosensors that also rely on antibodies as recognition elements for the detection of cTnI^22^,^23^. Considering the limited stability of antibodies, the translation of such assays for the rapid diagnosis in resource-limited settings is challenging. Having a simple, rapid and reliable diagnostic test that can be deployed in an ambulance, intensive care unit emergency room, or battlefield eliminates the time lapse associated with sample transport, log-in and assay, thus hastening therapeutic intervention.

In this paper, using BPDs as a sensing platform for cTnI, we investigate the sensitivity and stability of LSPR biosensors based on natural antibodies and short peptides, as BREs. We demonstrate that peptides provide a significantly higher sensitivity and lower limit of detection compared to antibodies as a recognition element. Furthermore, unlike antibodies, we show that peptides exhibit excellent stability by retaining their target-recognition capability after exposure of BPDs to elevated temperatures. The rapid detection of cTnI in biological fluids at clinically relevant concentrations using BPDs can be a robust approach for biodiagnostics in resource-limited settings.

## Results and Discussion

**Figure 2:**
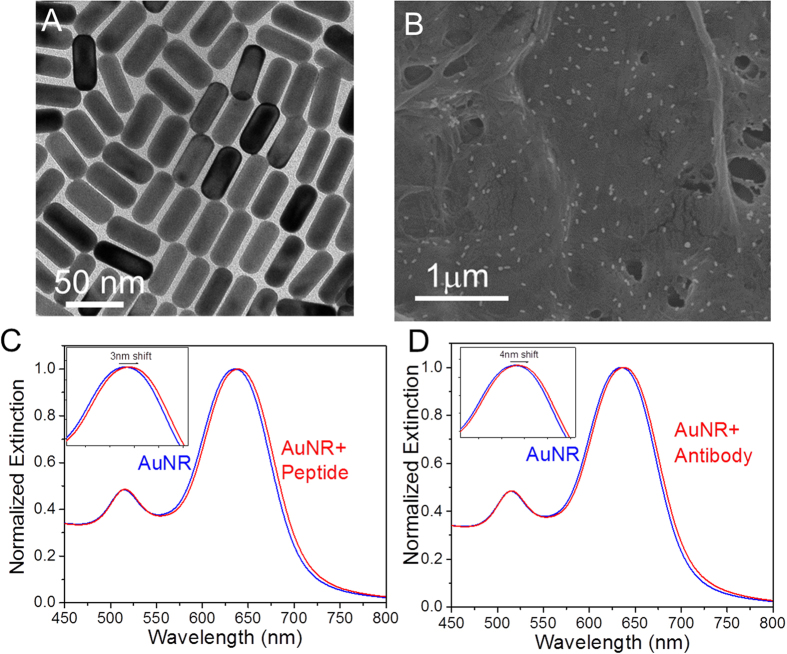
(**A**) TEM image of AuNRs used as plasmonic nanotransducers. (**B**) SEM image depicting the uniform adsorption of peptide-conjugated Au nanorods on paper substrate. Extinction spectra showing the LSPR shift after conjugation of AuNR with (**C**) troponin binding peptide and (**D**) anti-cTnI antibody. Insets show the magnified image of the shift.

Gold nanorods (AuNR) are an attractive choice as plasmonic nanotransducers for label-free sensing because of their geometrical tunability of LSPR wavelength and high sensitivity^24^. We synthesized AuNRs using a seed-mediated approach with a length of 47.3 ± 2.3 nm and a diameter of 20.2 ± 1.4 nm (Fig. 2A). The human troponin I binding peptide (-FYSHSFHENWPS-), identified from a phage display peptide library, with nanomolar (nM) binding affinity to cTnI was employed here as the biorecognition element^25^. The conjugation of the peptide to AuNRs as achieved by introducing a cysteine residue at the C-termini of the peptide (-FYSHSFHENWPSC-), to facilitate binding to AuNRs through an Au-S linkage^26^. We measured the kinetics of cTnI binding to the cysteine-modified peptide after its appendage to Au surface using quartz crystal microbalance (QCM) and found the dissociation constant (k_d_) to be 1.5 × 10^8^, which closely agrees with the value reported by Banta and co-workers (Supplemental Figure S1)^27^. After coupling the peptide to the AuNR surface, the LSPR wavelength of the AuNRs exhibited a red shift of ~3 nm due to the increase in the refractive index of the medium surrounding AuNR (Fig. 2C). The peptide conjugated AuNRs were then uniformly adsorbed on the paper substrate (Fig. 2B). The successful conjugation of the peptide to the surface of the AuNR was also confirmed using surface enhanced Raman scattering (SERS) spectroscopy. SERS spectra obtained from the paper substrates reveal Raman bands corresponding to C-C, C-N+ vibrations at 1004 cm^−1^ from phenylalanine and 1341 cm^−1^, 1360 cm^−1^ from tryptophan (Supplemental Figure S2)^28^,^29^.

As mentioned above, we sought to compare the performance of peptides vs. natural antibodies as recognition elements in BPDs. For this purpose, we used anti-cTnI (N-terminus), a polyclonal antibody, as the conventional antibody recognition element. Anti-cTnI was conjugated to the AuNR using a carbodiimide crosslinker chemistry and thiol-terminated poly (ethylene glycol) (SH-PEG) (see Experimental Section for details). The SH-PEG conjugation prevents the anti-cTnI from non-specifically binding to the AuNR surface, thus preventing the blocking of the cTnI-binding epitope (Supplemental Figure S3). Using a dot-blot dilution immunoassay, we confirmed that the affinity of the antibody towards troponin is preserved after bioconjugation with SH-PEG (Supplemental Figure S4). The pegylated anti-cTnI binds to the surface of the nanotransducer via an Au–S linkage. The coupling of anti-cTnI to AuNR resulted in a red shift of ~4 nm in the LSPR wavelength (Fig. 2D).

We have also used dynamic light scattering (DLS) to monitor the changes in the hydrodynamic size of the AuNR upon bioconjugation with cTnI binding peptide or anti-cTnI. The increase in the hydrodynamic radius in the case of the peptide-conjugated AuNR (~1.4 nm) is much smaller compared to that observed for the antibody-conjugated AuNR (~11.8 nm) due to the significantly smaller molecular weight of the peptide (~1.6 kDa) compared to the antibody (150 kDa) (Supplemental Figure S5). The dry state thickness of the peptide and antibody recognition layers was measured using atomic force microscopy (AFM; Supplemental Figure S6). The results suggest that the increase in the diameter of the AuNRs after antibody conjugation (~4.2 nm) was significantly higher compared to that after peptide conjugation (~1 nm) in their dried state.

We have recently introduced BPDs as a novel platform for LSPR-based biosensors^30^. Paper substrates offer numerous advantages such as high surface area, excellent wicking properties, low cost, easy disposability, small sample volume requirement, and facile processing (cutting, bending, dipping)^31^,^32^ The peptide- and antibody-conjugated AuNRs were adsorbed on a filter paper by the immersion of a 1 × 1 cm strip of filter paper in a solution of BRE-conjugated AuNR (Supplemental Figure S7A). The SEM image of the paper revealed a uniform distribution of the bioconjugated AuNRs with no signs of aggregation or patchiness (Fig. 2B and Supplemental Figure S7B). The areal density of the AuNR-antibody and AuNR-peptide conjugates adsorbed on the paper surface was found to be 52 ± 3/μm^2^ and 49 ± 2/μm^2^. The density of the AuNRs is quite similar in both cases thus minimizing any effects on the sensitivity due to variations in density.

We also verified that the affinity of peptide- and antibody-conjugated AuNR to cTnI is comparable. We performed SDS-PAGE using peptide and antibody-conjugated AuNRs and blotted with polyclonal goat antibody (Supplemental Figure S8). We found that the affinities of the AuNRs conjugated with both BREs (antibody and peptide) were almost identical. We also compared the affinity of peptide- and antibody-conjugated AuNR to cTnI using anti-mouse IgG-horse radish peroxidase (HRP) conjugate. The colorimetric assay was implemented using non-overlapping monoclonal antibody to cTnI, anti-IgG-HRP conjugate and 3,3′,5,5′-tetramethylbenzidine (TMB-8) as the substrate. The colorimetric assay enabled us to assess the amount of cTnI bound to the biofunctionalized AuNR under saturation condition. The absorbance at 450 nm, which is indicative of cTnI bound to bioconjugated AuNR, in the case of peptide conjugated AuNR was comparable to that of the antibody conjugated AuNR as shown in Supplemental Figure S8.

The extinction spectra collected from several spots across the surface of the paper (1 × 1 cm) showed excellent optical uniformity with a standard deviation of <1 nm in longitudinal wavelength (Supplemental Figure S9A). The extinction spectrum from the paper is collected from a 2 μm^2^ area, which corresponds to ~200 nanorods, using a microspectrometer mounted on an optical microscope. Each extinction spectrum has the baseline subtracted and is deconvoluted using a two peak Gaussian fit (Supplemental Figure S9B). The concentration of the AuNR solution employed for the adsorption of bioconjuagted AuNR onto paper is critical in order to achieve an optically homogeneous sensing substrate which is critical in the design of a biosensor. Optical uniformity defines the noise level of a biosensor and thus determines the limit of detection (LOD). The longitudinal LSPR is used for detecting and monitoring the target binding events because of its higher refractive index sensitivity compared to the transverse LSPR band^33^. After the absorption of AuNR on paper, the LSPR wavelength of AuNR exhibited a blue shift of ~17 nm compared to that in solution due to a decrease in the effective refractive index of the surrounding medium from water to air and paper substrate. (Supplemental Figure S10)^34^.

**Figure 3:**
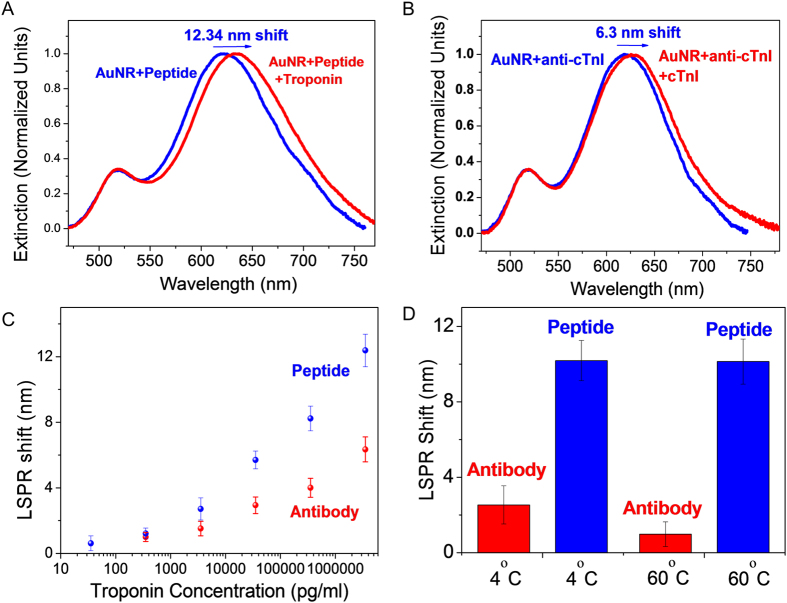
Extinction Spectra showing LSPR shift after cTnI binding with (**A**) peptide conjugated AuNR (**B**) antibody conjugated AuNR. (**C**) Troponin sensing of antibody and peptide conjugated AuNR at different concentrations. (**D**) LSPR shift after exposure to the cTnI (3.53 μg/ml) for antibody and peptide conjugated AuNR after 48 hr incubation at 4 °C and 60 °C.

To probe the sensing capability of the BPDs, we exposed the BPDs to troponin I (3.53 μg/ml in 0.1 M TBS pH 8.0). The extinction spectra obtained from the peptide-AuNR and antibody (anti-cTnI)-AuNR BPDs showed a red shift of 12.3 nm and 6.3 nm, respectively (Fig. 3A,B). The higher sensitivity of peptide-AuNR compared to antibody-AuNR is due to differences in the thickness of the peptide and antibody adsorbate layer as will be discussed in detail below. To probe the limit of detection in both cases, BPDs with bioconjugated AuNR were exposed to varying concentrations of cTnI. In both cases, a monotonic increase in the LSPR shift was observed with an increasing concentration of the cTnI (Fig. 3C). The limit of detection for peptide-based BPDs was found to be ~35.3 pg/ml, which is an order of magnitude lower than that of antibody-based BPDs (~353 pg/ml). To compare the selectivity of the peptide and antibody towards cTnI, we used human serum albumin (HSA) as an interfering protein (Supplemental Figure S11). Both antibody and peptide-modified AuNR exhibited small LSPR shifts upon exposure to high concentrations of HSA demonstrating the excellent selectivity of the bioconjugated AuNR to cTnI.

Yet another significant advantage of utilizing peptides as recognition elements compared to antibodies is the enhanced chemical and environmental stability of peptides compared to antibodies. As opposed to antibodies, which denature and loose molecular recognition ability at elevated temperatures, absence of a secondary structure in peptide recognition elements renders excellent stability even under harsh conditions. To probe the stability of BPDs based on peptides or antibodies, we incubated BPDs adsorbed with bioconjugated AuNR at 4 °C and 60 °C for 48 hr. Subsequently, the performance of BPDs was tested by evaluating for the detection of cTnI. The peptide-based BPD exhibited remarkable thermal and temporal stability by exhibiting a consistent LSPR shift. On the other hand, antibody-based BPD showed degradation in performance, as evidenced by smaller LSPR shift, after exposure to elevated temperatures. Even the BPDs stored at 4 °C for 48 hours showed significantly smaller LSPR shift compared to freshly prepared paper substrates due to the poor shelf-life stability of antibodies in dry state (Fig. 3D). This remarkable temperature and shelf-life stability makes peptide-based BREs excellent candidates for LSPR-based point-of-care diagnostics in resource-limited settings.

**Figure 4:**
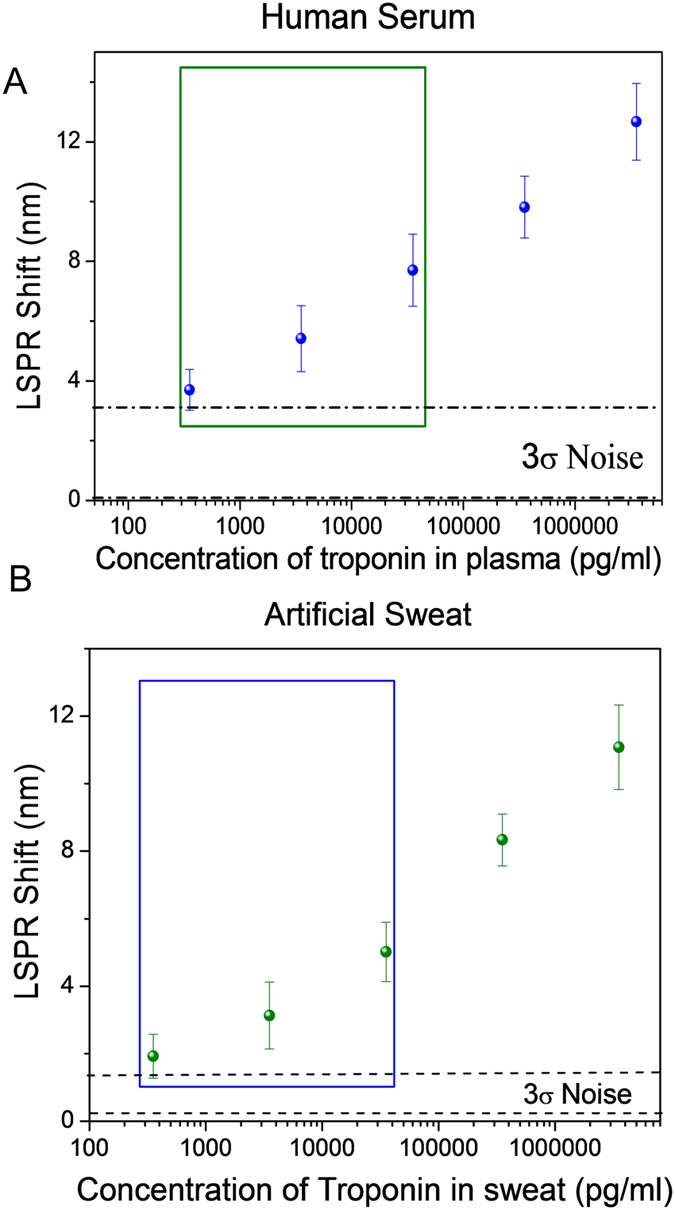
(**A**) Sensing calibration curve of cTnI spiked in human plasma (1/10^th^ concentration) in buffer. (**B**) Sensing calibration curve of cTnI spiked in artificial sweat (1/10^th^ concentration) in buffer. The boxed data points represent the physiologically relevant concentration of cTnI detection levels (~ng/ml concentration) over the 3σ noise level.

Considering the high sensitivity and shelf-life stability of peptide-based BPDs, we next sought to detect troponin in complex physiological fluids such as human plasma and sweat. Cardiac troponins in blood are considered to be the most sensitive and specific biomarkers for myocardial infarction, a medical condition that involves myocardial tissue injury^35^. We employed peptide-conjugated AuNR BPDs to detect troponin in 10% human plasma. To reduce non-specific binding, the BPDs were exposed to 1% HSA to block the non-specific binding sites (see Experimental Section for details). The LSPR shift exhibited a monotonic increase with increase in the concentration of troponin in human plasma and artificial eccrine sweat (Fig. 4). The limit of detection of cTnI in human plasma was 353 pg/ml is within physiologically relevant concentration range of troponin in human plasma, 0.010–10 ng/ml^36^, our BPDs demonstrated here provide a means for ultrasensitive detection of cTnI. Furthermore, we have demonstrated that troponin can also be detected in artificial eccrine sweat at physiologically relevant concentrations (Fig. 4B).

**Figure 5:**
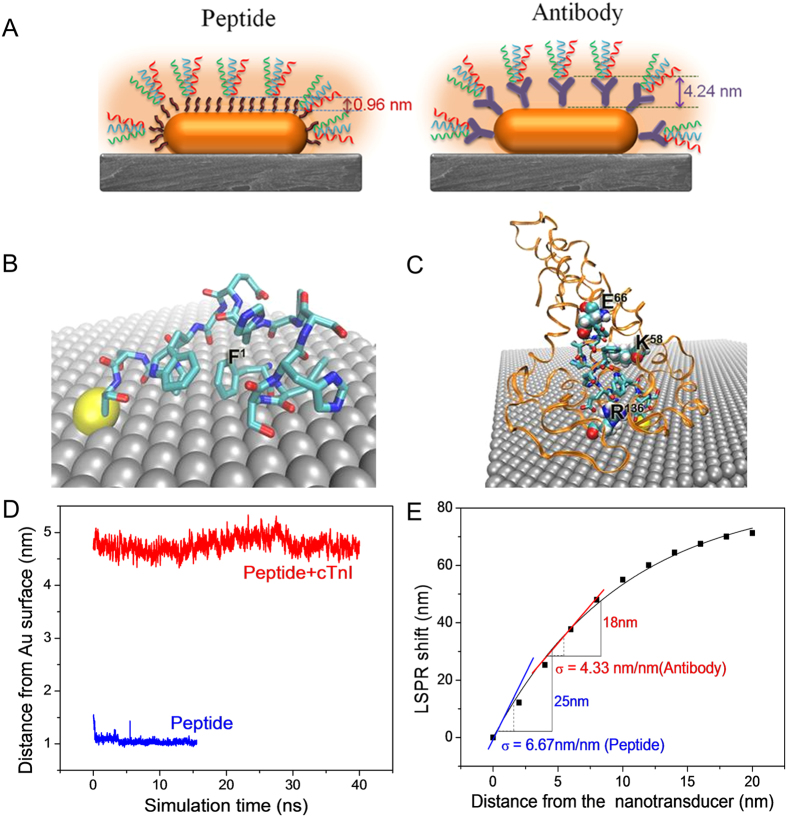
(**A**) Schematic showing the effect of the distance of the peptide or antibody recognition element from the surface of the nanotransducer on the refractive index sensitivity. (**B**) Predicted peptide structure on the gold surface. The amino-termini phenylalanine (F1) is shown. (**C**) Computationally predicted structure of peptide binding to cTnl on the gold surface. Key residues of the cTnI binding pocket are labeled (**D**) The time evolution of adsorbate thickness on the gold surface. (**E**) Distance dependent sensitivity (σ) of AuNRs adsorbed on a glass substrate showing different σ for antibody and peptide. The σ values obtained from the curve for peptide is 6.67 nm/nm and antibody is 4.33 nm/nm. The corresponding shift for peptide from this curve is 24.5 nm whereas from antibody is 18 nm.

Now we turn our attention to the structure-based functional mechanism of the peptide-conjugated BPDs. The structure and thickness layer of the peptide recognition element on the gold surface was also investigated using molecular dynamic simulations (described in the supplemental section). The atomistic structure of the cTnI binding peptide shows that N-terminal Phe^1^ and C-terminal Cys^13^ residues are in close contact with the gold surface (Fig. 5B). The amino acid Trp^10^ in the peptide (a possible binding residue to cTnI) is distal to the gold surface and accessible for cTnI binding. The three key amino residues in the binding pocket of cTnl are Lys58, Glu66 and Arg136 depicted in Fig. 5C are able to non-covalently interact with the cTnI binding peptide on the surface of the gold. These key residues provide structural information and will be used in further studies for the optimization of peptide recognition elements for cTnI. Of importance to this study, the thickness of the biomolecule adlayer can be obtained from our molecular dynamic simulations. As discussed above, AFM imaging revealed the thickness of the peptide layer (0.96 nm) on AuNR to be significantly smaller than that of the antibody (4.24 nm) (Fig. 5A). The smaller thickness of the peptide layer renders higher sensitivity of BPDs using peptide recognition elements. For calculating the thickness layer from our computational models, the gold surface is aligned in the XY plane and the thickness of the peptide or peptide-cTnI complex on the gold surface is calculated as the maximum difference of the Z-coordinates. Figure 5D shows the time evolution of the thickness of the peptide layer and the peptide-cTnl complex. As shown in Fig. 5D, the saturated values is 1.03 ± 0.03 nm for the peptide layer and 4.74 ± 0.11 nm for the peptide-cTnl complex on the gold surface. AFM measurements of the peptide conjugated AuNR after cTnI binding revealed that the diameter of AuNR increased by ~3.8 nm (Supplemental Figure S12). Adding the thickness of peptide in dry state (~0.9 nm), the thickness of the protein on the surface of the nanorod is ~4.8 nm, which is in excellent agreement with that predicted by the MD simulations shown in Fig. 5D.

We probed the distance-dependent sensitivity of the AuNRs, nanotransducers used in this study, by depositing polyelectrolyte multilayers using layer-by-layer (LbL) assembly. Extinction spectra were collected after the deposition of each bilayer comprised of poly(allylamine hydrocholoride) (PAH) as a positively charged polymer and poly(styrene sulfonate) (PSS) as a negatively charged polymer. As described above, the LSPR wavelength shift exhibited a characteristic decay with the increasing distance from the surface of the nanorods (i.e., increasing number of polyelectrolyte layers). It is instructive to define a parameter called distance-dependent refractive index sensitivity (σ), which is the LSPR shift caused by the deposition of a 1 nm thick dielectric layer (polyelectrolyte multilayers in the present case) at a predetermined distance from the surface of the nanotransducer. Such a parameter can be easily deduced at different distances from the AuNR surface from the plot shown in Fig. 5E. The extinction measurements after troponin binding to the bioconjugated AuNR were performed in the dry state. So, considering that the thickness of peptide and antibody in their dry state is ~1 nm and ~4.2 nm for peptide and antibody, respectively, the values of σ are computed as shown in Fig. 5E. For peptide recognition elements, σ is found to be 6.67 nm/nm whereas in case of antibody, σ is found to be 4.33 nm/nm. From the distance-dependent refractive index sensitivity measurements, it can be seen that the local refractive index sensitivity with peptide recognition elements is nearly 50% higher than compared to that with antibody-based recognition elements. Our experimental observations indicate nearly 100% higher LSPR shift (at the highest concentration tested here 3.53 μg/ml) with peptides compared to antibodies as recognition elements. This discrepancy can possibly be due to the higher number of cTnI molecules bound on the AuNR surface in the case of the peptide recognition elements compared to antibodies. The low steric hindrance of the peptides facilitates a higher density of the recognition elements on the surface of the Au nanotransducers and thus a higher density of cTnI molecules. This observation will be further explored in future studies in order to maximize the sensitivity of our BPDs.

In summary, we have demonstrated that, in label free plasmonic biosensing, the affinity of the recognition element can be overpowered by the distance-dependent sensitivity (σ) of the biosensor. We further showed that peptides are excellent candidates as recognition elements for LSRP-based sensing. The BPDs described here can be easily adapted to other biomarkers of interest by functionalizing the AuNRs with the appropriate BREs. Improvements in the binding affinity of peptide-based BREs will have to be pursued in order to achieve a lower limit of detection and a higher selectivity in order to compete to natural antibodies. Multiplexed BPDs can be developed to improve sensitivity and detection but also enable detection of multiple biomarkers by fabrication of multi-channel microfluidic paper-based devices^37^,^38^. Furthermore, the BPDs can be easily implemented for application in resource-limited or austere settings such as in remote locations and battlefield settings using a simple, low-cost handheld vis-NIR spectrometer in either transmission or reflection mode.

## Experimental Section

### Material

Cetyltrimethylammonium bromide (CTAB), chloroauric acid, ascorbic acid, sodium borohydride, tris(hydroxymethyl)amino methane (tris), human Serum albumin (HSA), poly(stryrene sulfonate) (PSS) (Mw = 70,000 g/mol) and poly(allyl amine hydrochloride) (PAH) (Mw = 56,000 g/mol) were purchased from Sigma Aldrich. Silver nitrate and filter paper (Whatman #1) was purchased from VWR international. 1-Ethyl-3-(3-dimethylaminopropyl) carbodiimide (EDC) and N-hydroxy succinimide(NHS) were purchased from Thermo Scientific. SH-PEG-COOH (Mw = 5000 g/ml) was purchased from Jenkem Technology. Antibodies against human cardiac troponin I (H-41) was obtained from Santa Cruz Biotechnology. Concentrated phosphate buffer saline (PBS) 10X was purchased from Omnipur. The troponin binding peptide was purchased fron Genscript USA Inc., Human Cardiac Troponin- I Recombinant was purchased from Life Diagnostics, USA. Artificial eccrine was purchased from Pickering Laboratories. All chemicals have been used as received with no further purification.

### Synthesis of gold nanorods (AuNRs)

Gold nanorods were synthesized using a seed-mediated approach^39^. Seed solution was prepared by adding 0.6 ml of an ice-cold sodium borohydride solution (10 mM) into 10 ml of 0.1 M cetyltrimethylammonium bromide (CTAB) and 2.5 × 10^−4^ M chloroauric acid (HAuCl_4_) solution under vigorous stirring at 25 °C. The color of the seed solution changed from yellow to brown. Growth solution was prepared by mixing 95 ml of CTAB (0.1 M), 0.5 ml of silver nitrate (10 mM), 4.5 ml of HAuCl_4_ (10 mM), and 0.55 ml ascorbic acid (0.1 M) consecutively. The solution was homogenized by gentle stirring. To the resulting colorless solution, 0.12 ml of freshly prepared seed solution was added and set aside in the dark overnight. Prior to use, the AuNRs solution was nanopure water (18.2 MΩ·cm).

### AuNR-Peptide conjugation preparation

AuNR-peptide conjugates were prepared by adding 8 μl of the peptide (concentration 1.31 mM in water), 2 μl at a time to a solution of 1 ml of twice centrifuged nanorods. The solution was left overnight on a shaker to homogenize the conjugation. The resulting nanorod-peptide conjugates showed a shift of ~3 nm.

### AuNR-Troponin H-antibody conjugation preparation

A solution was prepared by adding 67 μl of heterobifunctional polyethylene glycol (SH-PEG-COOH) in water (2 μM) with the same molar ratio of EDC and NHS, followed by shaking for one hour. The pH of the above reaction was adjusted to 7.4 by adding 10 × concentrated PBS, followed by the addition of 100 ul of Troponin H-terminus antibody (1.34 μM, Mw = 150 kDa). The reaction mixture was incubated for an additional two hours and filtered to remove excess chemicals and byproducts by centrifugation using a centrifuge tube with 50 kDa filter. The conjugate mixture is obtained by washing the conjugates with water twice. The AuNR-cojugate mixture was obtained by adding 6 ul of SH-PEG-antibody conjugate of concentration 1.34 μM to 1 ml of twice centrifuged nanorods. Using SDS-PAGE, we confirmed that the affinity of the SH-PEG-antibody is the same as pristine antibody.

### Bioplasmonic paper substrate preparation

A regular laboratory filter paper (Whatman #1) was used for the absorption of nanorods. A 1 cm × 1 cm paper strip is taken and immersed in a solution of AuNR conjugates (peptide and antibody) and left overnight at 4 °C. The paper strip was taken out and washed with tris buffer and immersed in different concentrations of troponin in tris buffer (pH 8) for 2 hours at 4 °C. It was removed and washed thoroughly with tris buffer and dried under a stream of nitrogen.

### Spiked Human Serum and Artificial Sweat test

In case of testing the spiked human serum, samples were exposed to 1% Human Serum Albumin to saturate the non-specific binding sites on the paper substrate prior to troponin exposure. For testing the biosensor, troponin of various concentrations was used to spike human serum (1/10^th^ concentration) in 0.1 M TBS (pH 8). Similarly, troponin of various concentrations was used to spike artificial sweat) (1/10^th^ concentration) in tris buffer (pH 8). Bioplasmonic paper was immersed in these solutions and left at 4 °C for two hours. It was removed and washed thoroughly with tris buffer to remove all the non-specific binding.

### Extinction spectra measurements

Extinction spectra from paper substrates were collected using a CRAIC microspectrophotometer (QDI 302) coupled to a Leica optical microscope (DM 4000 M) with 20× objective in the range of 450–800 nm with 10 accumulations and 100 ms exposure time in reflection mode. The spectral resolution of the microspectrophotometer is 0.2 nm. Multiple UV-Visible spectra (~10) were collected from different locations of the paper strip before and after exposure to troponin solution. Shimadzu UV-1800 spectrophotometer was employed to collect UV-Vis extinction spectra from solution.

### Layer-by Layer Assembly

Glass substrates were modified by 1% P2VP followed by the adsorption of AuNR. For layer-by-layer assembly, AuNR substrates were immersed in 1 wt% PSS in 0.1 M NaCl aqueous solution for 15 min followed by rinsing with DI-H_2_O water for 30 s and rinsing with 0.1 M NaCl solution for an additional 30 s on each side of the glass slides. Then the substrates were immersed in a solution of 1 wt% PAH in 0.1 M NaCl for 15 min followed by the rinsing procedure described above. Subsequently, the substrates were dried by nitrogen stream before obtaining extinction spectra with an UV−vis spectrometer. Procedure mentioned above was repeated 10 times to deposit a total of 10 bilayers. The thickness of each polyeletrolyte bilayer was ~2 nm.

### Characterization

Transmission electron microscopy micrographs were recorded on a JEM-2100F (JEOL) field emission instrument. Samples were prepared by drying a drop of the solution on a carbon coated grid, which had been previously made hydrophilic by glow discharge. Scanning electron microscope images were obtained using a FEI Nova 2300 Field Emission SEM at an accelerating voltage of 10 kV. The paper was gold sputtered for 60 second before SEM imaging.

### Construction of the Molecular Models

The gold surface of size 14.1 × 14.5 × 0.7 nm^3^ was made using Materials Studios. The peptide and the cTnl protein (template PDB bank ID 1J1E) were constructed using I-TASSER^40^. The terminal ends were capped by the acetyl (ACE) and the N-methylamide (NME) groups. The bond S-Au was modeled using a harmonic potential 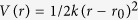 where 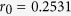 *nm* and *k *= 82843.2 *kJ*/*mol*/*nm*^2^ 41. AMBER 99SB force field was used to describe the peptide and the cTnl protein^42^. FFB Van der Waals parameters were chosen to describe the Au-Au interactions based on a recent test^41^. The structure of the peptide on the gold surface was first predicted using the following protocol of molecular dynamics simulations. Then the cTnl protein was docked on the peptide regarding the peptide as fixed using ZDOCK^43^. Several docked configurations were refined using the following molecular dynamics methods. The lowest energy configuration was further refined using replica exchange molecular dynamics simulations. All structural images were produced using VMD^44^.

### Molecular Dynamics Simulations

To mimic the dry states in our experiments, all molecular dynamics simulations were carried out in vacuum using NAMD^45^. Periodic boundary conditions were used so that infinite size of gold surface was modeled. In the direction perpendicular to the gold surface, 30 nm empty spaces were imposed to avoid interactions with images. A cutoff distance 1.2 nm with a smooth switching function applied beyond 1.0 nm was used for short-range interactions. A constant temperature 300 K was maintained. The particle-mesh Ewald was used to calculate the long-range electrostatic interactions. A system was first minimized for 2000 steps. Then it was gradually heated up to 300 K and allowed to equilibrate. The obtained structures were further refined using replica exchange molecular dynamics simulation. Eight replicas were used with temperature ranged between 277.15 K and 333.15 K to meet our experimental interest.

## Additional Information

**How to cite this article**: Tadepalli, S. *et al.* Peptide Functionalized Gold Nanorods for the Sensitive Detection of a Cardiac Biomarker Using Plasmonic Paper Devices. *Sci. Rep.* **5**, 16206; doi: 10.1038/srep16206 (2015).

## Supplementary Material

Supplementary Information

## Figures and Tables

**Figure 1:**
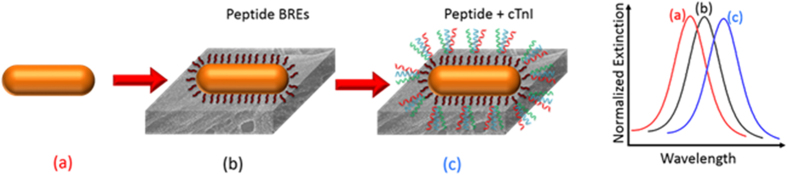
Schematic representing the design of the biosensor with peptide recognition elements. (**a**) AuNR (**b**) AuNR + peptide BREs (**c**) AuNR + peptide BRE + cTnI.
