# Supplementary material for: Peptide Functionalized Gold Nanorods for the Sensitive Detection of a Cardiac Biomarker Using Plasmonic Paper Devices
Source: Sci Rep. 2015 Nov 10;5:16206. doi: 10.1038/srep16206 (PMC4639779; doi:10.1038/srep16206)
Supplement: Supplementary Information [file srep16206-s1.doc]

**Supporting Information:** **Peptide Functionalized Gold Nanorods for the Sensitive Detection of a Cardiac Biomarker Using Plasmonic Paper Devices**

Sirimuvva Tadepalli^1^, Zhifeng Kuang^2^, Qisheng Jiang^1^, Keng-Ku Liu^1^, Marilee A. Fisher^3^, Jeremiah J. Morrissey^3,4^, Evan D. Kharasch^3,4,5^, Joseph M. Slocik, Rajesh R. Naik^2*^ , Srikanth Singamaneni^1,4*^

*^1^Institute of Material Science and Engineering and Department of Mechanical Engineering and Material Science, Washington University in St. Louis, St Louis, MO, 63130, USA.*

*^2^Soft Matter Materials Branch, Materials and Manufacturing Directorate, Wright Patterson Air Force Base, Dayton, OH 45433, USA*

*^3^Department of Anesthesiology, Division of Clinical and Translational Research, Washington University in St. Louis, St. Louis, MO 63110, USA*

*^4^Siteman Cancer Center, Washington University in St. Louis, St. Louis, MO 63110, USA*

*^5^Department of Biochemistry and Molecular Biophysics, Washington University in St. Louis, St. Louis, MO 63110, USA*

***To whom correspondence should be addressed:** [**rajesh.naik@us.af.mil**](mailto:rajesh.naik@us.af.mil) **(RRN)** [**singamaneni@wustl.edu**](mailto:singamaneni@wustl.edu) **(SS)**


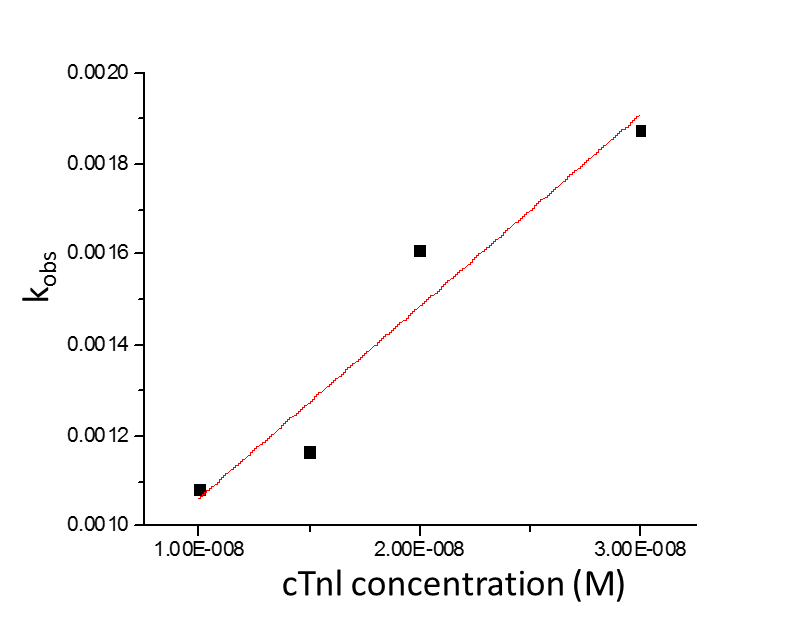


**Figure S1:** Kinetics of the binding of cTnI to the peptide on the surface of Au as determined by quartz crystal microbalance. **Figure S2:** Raman spectrum taken from the paper substrate confirming the peptide conjugation on the AuNR surface.


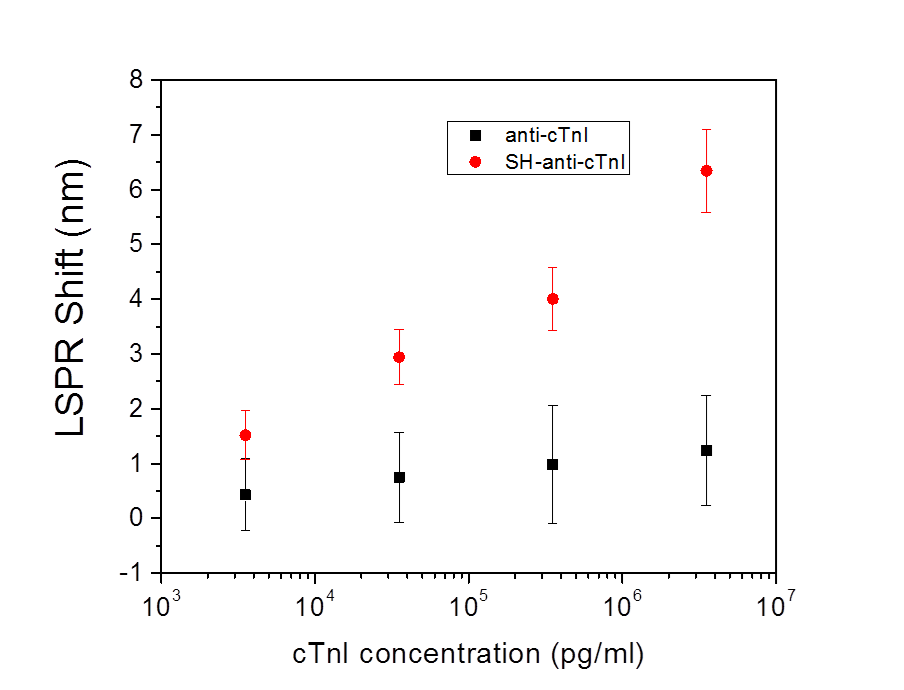


**Figure S3**: LSPR shift of cTnI binding to antibody conjugated to AuNR comparing the affinity of antibody conjugated through SH-PEG to AuNR Vs the antibody that is non-specifically bound on the AuNR.


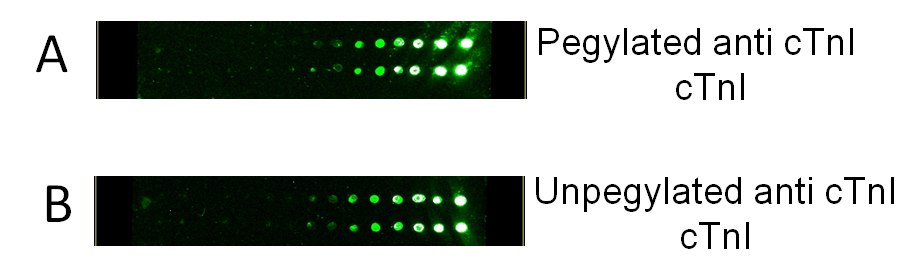


AA

BA

**Figure S4:** Dot-blot dilution of recombinant human cardiac troponin I (cTnI) using anti-cTnI confirms that the affinity towards cTnI in (A) Pegyated and (B) Unpegylated antibody. Antigen-antibody intearction was not affected by bioconjugation with EDC-NHS. cTnI concentrations 350, 175, 35, 17.5, 3.5, 1.75, 0.35, 0.175, ng right to left.

**Figure S5:** Hydrodynamic Radius of AuNRs showing an increase in the hydrodynamic size after peptide and antibody conjugation.


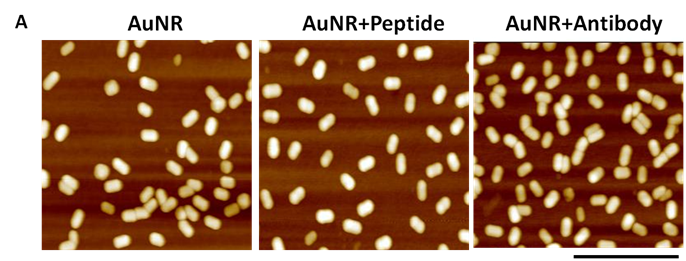


AA

BA


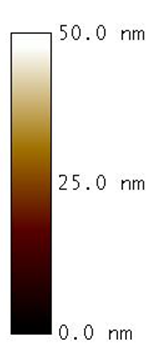


**Figure S6:** (A) AFM images of the AuNR, AuNR+peptide and AuNR+antibody. Scale bar represents 500nm. (B) Height profile of the AuNR following successful bioconjugation showing the difference in the height of the recognition element.

B**A**BA

AA


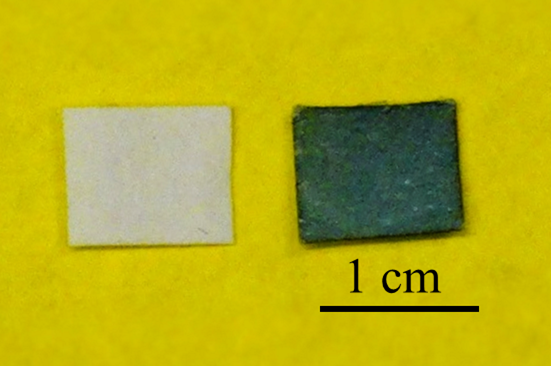

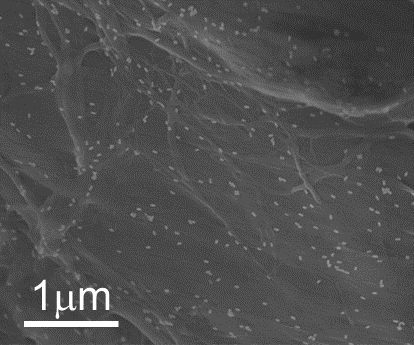


**Figure S7:** (A) Peptide Conjugated AuNR adsorbed on paper (B) SEM image showing the uniform distribution of antibody conjugated nanorods on the fibers of paper.


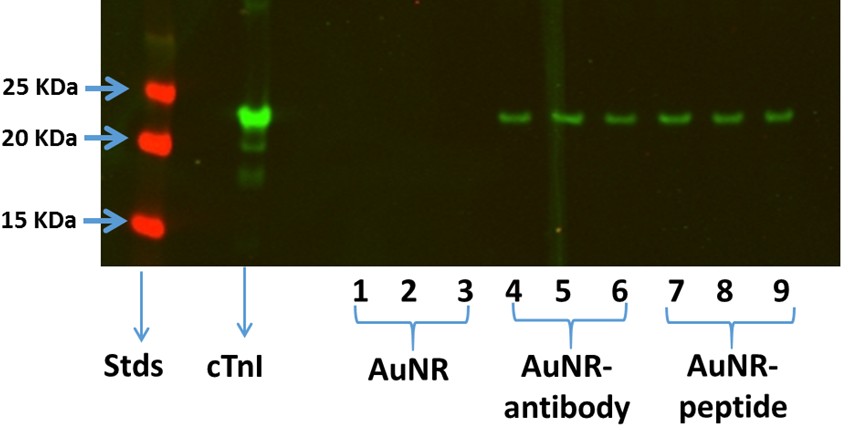

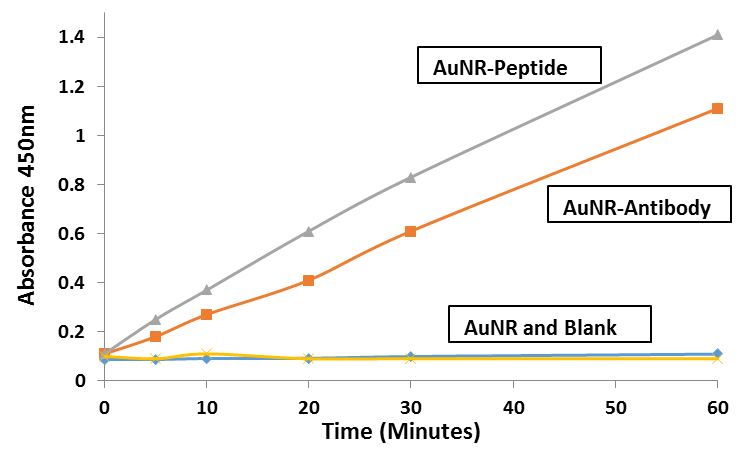


B**A**BA

AA

**Figure S8**: (A) SDS-PAGE with antibody and peptide conjugated with AuNR by blotting with polyclonal goat antibody showing similar affinity of peptide and antibody BREs. (B) TMBA assay comparing the cTnI concentration with monoclonal antibody to cTnI and anti-mouse IgG-HRP conjugate.

B**A**BA

AA

**Figure S9:** (A) Extinction Spectra of peptide conjugated nanorods after absorption on paper at different locations showing a homogenous LSPR with a standard deviation of less than 1nm. (B) A representative LSPR spectrum from the paper substrate deconvoluted using a two peak Gaussian fit.

B**A**BA

AA

**Figure S10:** UV-Vis spectra showing a blue shift in the LSPR peak position of (A) Peptide (B) Antibody conjugated nanorods after adsorption on paper because of the refractive index change.

**Figure S11:** LSPR shift at different concentrations of Troponin and Human Serum Albumin showing that the selectivity of antibody is better than that of the peptide.

**Figure S12**: AFM thickness profile AuNR with peptide-CnTI complex.
